# Diagnostic performance of one visual aura image in identifying migraine with aura

**DOI:** 10.3389/fneur.2026.1774218

**Published:** 2026-03-06

**Authors:** Nancy van Veelen, Estelle van Eijk, Irene de Boer, Gisela M. Terwindt

**Affiliations:** Department of Neurology, Leiden University Medical Center, Leiden, Netherlands

**Keywords:** aura, clinic, diagnostic, image, migraine

## Abstract

**Background:**

Accurate diagnosis of migraine with aura is crucial. This study compared diagnostic accuracy of a visual aura image alone and the same image combined with additional questions, using the clinical diagnosis of migraine with or without aura as the reference standard.

**Methods:**

In this cross-sectional diagnostic accuracy study conducted by the Leiden Migraine Group between September 2024 and January 2025, patients with diagnosed migraine were recruited. Upon enrolment, participants completed a survey followed by a questionnaire featuring an image depicting a typical visual aura disturbance. Only one image was used, as presenting multiple images may be impractical in clinical settings or large epidemiological studies.

**Results:**

A total of 481 migraine patients were included, 171 with aura and 310 without aura. The visual aura image alone demonstrated a sensitivity of 0.89 (95%CI 0.84, 0.94), a specificity of 0.65 (0.60, 0.70), a positive predictive value (PPV) of 0.59 (95%CI 0.52, 0.65), and a negative predictive value (NPV) of 0.92 (95%CI 0.87, 0.95). Adding a validated extensive questionnaire reduced sensitivity to 0.53 (95%CI 0.45, 0.60), but increased specificity to 0.90 (95%CI 0.86, 0.93), with a PPV of 0.74 (95%CI 0.65, 0.81) and NPV of 0.77 (95%CI 0.73, 0.82).

**Conclusion:**

A single visual aura image identifies most migraine with aura cases but shows limited specificity, frequently misclassifying patients without aura. Adding structured questions about visual symptom spread, positive phenomena, lateralization, duration, and timing relative to headache improves diagnostic accuracy, indicating that a visual aura image alone has limited clinical utility.

## Highlights

- One single visual aura image shows high sensitivity but limited specificity in individuals with migraine, leading to potential misclassification of patients without aura.
- For an accurate visual aura diagnosis, additional questions are required, particularly regarding gradual spreading and presence of positive phenomena, duration and unilaterality of aura, and temporal relation to headache.

## Introduction

Migraine with aura (MA) compared to migraine without aura (MO) is more strongly associated with various comorbidities, including ischemic stroke. Imaging studies have revealed distinct structural and functional brain alterations in MA, and specific genetic components are thought to contribute to its unique phenotype (1, 2). In addition, MA may respond differently to both acute and preventive treatment (3, 4). Distinguishing MA from MO remains challenging. A possible solution might be using visual aura iconography. Recently, there has been increasing interest in capturing and standardizing visual phenomena reported by patients with MA using detailed illustrations and image-based representations (5). Such visual depictions may enhance recognition of a visual aura among individuals with migraine. Although iconography of visual aura has been developed to describe various elementary visual symptoms, it has not yet been applied to differentiate between MA and MO (5). A potential limitation of iconography is the lack of detailed features, such as the spreading of symptoms, distinction between positive versus negative features, and their temporal relationship to headache onset (6).

This study examined whether a single, representative image of a typical visual aura, available online, could distinguish between MA and MO. For simplicity and clinical feasibility, we chose to use only one image, as our primary interest was to determine whether presenting a single visual aura image during consultation or for a large epidemiological or genetic study could aid recognition or if additional information is necessary for accurate diagnosis. We hypothesized that patients could recognize visual aura symptoms from the image and that combining it with a validated migraine-specific questionnaire would improve diagnostic accuracy. The study evaluated how accurately an image alone, and in combination with a questionnaire, distinguished between MA or MO, using a clinical diagnosis as the reference standard.

## Methods

### Design

The Leiden Headache Center (LHC) with the Leiden Migraine Group conducted a cross-sectional diagnostic accuracy study. Patients with a verified clinical migraine diagnoses from the Leiden Migraine Database were included. Data were collected between September 2024 and January 2025. The study was approved by the Leiden University Medical Center Medical Ethical Committee (24–3,013) and all participants provided digital informed consent.

### Participants

The Leiden Migraine Database is a cohort of Dutch adults with validated migraine diagnoses (with or without aura) according to the International Classification of Headache Disorders third edition (ICHD-3 criteria) (6, 7). This cohort includes both self-enrolled participants via web-based recruitment and patients from the Leiden Headache Center outpatient clinic, representing a broadly representative sample.

Eligible participants were ≥18 years and proficient in Dutch. Upon entry into the database, participants completed a validated electronic headache questionnaire, which applied an algorithm based on ICHD-3 criteria to classify patients as MO or MA (8). The questionnaire assessed visual aura characteristics as defined by the ICHD-3, including aura duration, timing relative to headache onset, lateralization, gradual spreading of symptoms and the presence of positive phenomena (e.g., scintillating lines, stars, flashing lights, colored spots, trembling air sensations, “wet windows glass,” loss of vision and diplopia).

**Figure 1:**
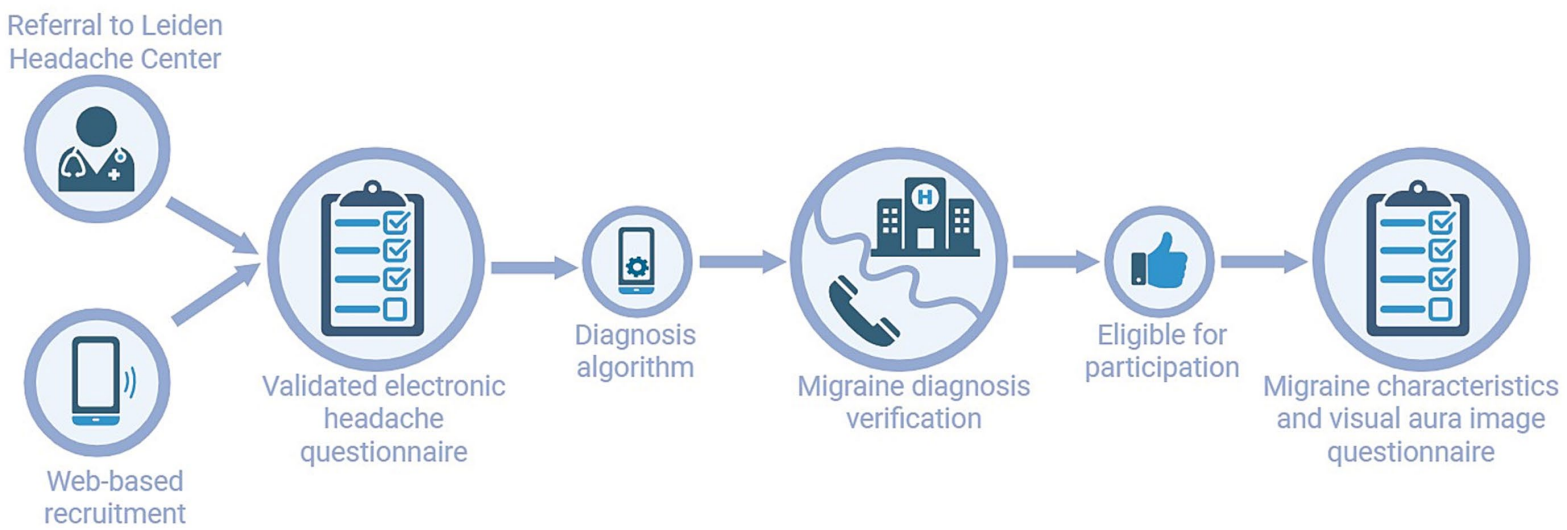
Study flow.

**Figure 2:**
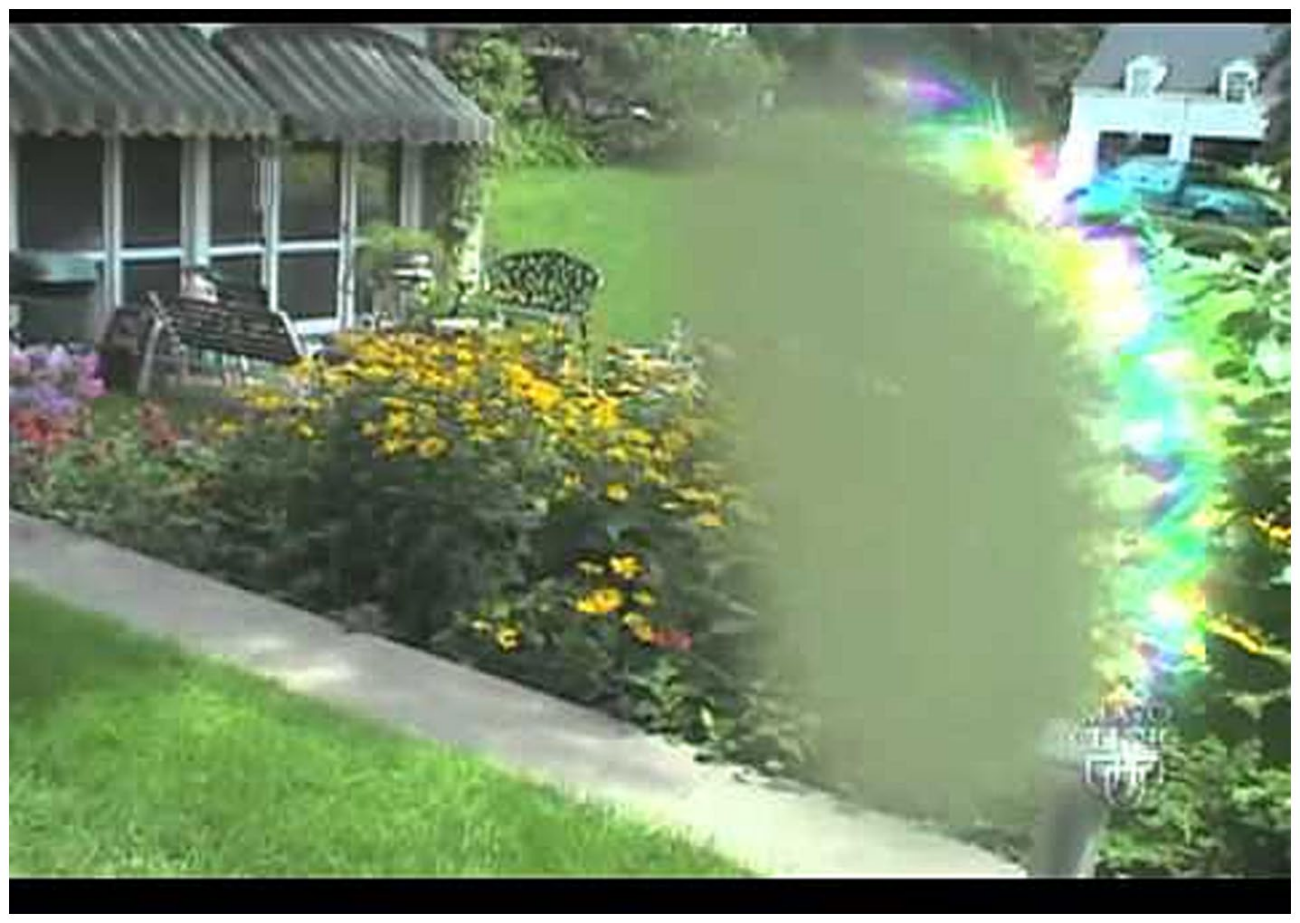
Representative image of a visual migraine aura shown to participants. Courtesy: Mayo Clinic, USA.

Each participants’ migraine diagnosis was subsequently verified by a medical doctor trained in headaches, in consultation with a neurologist specialized in headache (GMT), either during an in-person visit to the Leiden Headache Center or via telephone screening. Participants with verified migraine diagnosis were then eligible for the study and were sent a second questionnaire covering migraine characteristics, medication use, and questions on the presence of visual auras accompanied by a representative image (Figures 1, 2). This image, adapted from a visual aura illustration developed by the Mayo clinic, represents general visual aura features. Specifically, it depicts a scintillating scotoma, characterized by a central area of blurry or ‘foggy’ vision bordered by an arc of shimmering, multi-colored zig-zag lines and jagged fortification spectra. This image was chosen by expert opinion. Participants were asked: “Have you ever had a visual migraine aura? This image is an example of what a migraine aura can look like”. The answer to this question was yes or no. The image was an example the visual aura, the experiences aura did not need to be similar to the provided image. To evaluate the incremental value of the visual stimulus, physicians verifying the migraine diagnosis were provided with the results of the validated questionnaire but remained blinded to the participants’ responses regarding the visual aura image.

### Statistical analysis

Baseline characteristics were summarized as proportions, means with standard deviations, or medians with interquartile ranges, depending on data type. Differences between participants with MA and MO were assessed using an unpaired *t*-test for continuous variables, a chi-square test for categorical data, and a Mann–Whitney U test for non-normally distributed data.

Only participants who completed both the web-based form and the validated questionnaire at entry were included in the analysis. The verified medical diagnosis served as golden standard. Sensitivity, specificity, positive predictive value (PPV) and negative predictive value (NPV) were calculated with 95% confidence intervals (CI) for the visual aura image alone and for the combination of the visual aura image and the validated questionnaire. In the latter, participants were classified as MA only if both methods confirmed the diagnosis. The sensitivity and specificity of the two diagnostic tools were compared using McNemar’s chi-square test for paired proportions in the same cohort of participants. The comparisons of PPV and NPV were descriptive and not statistically tested, as these metrics are prevalence-dependent. *P*-values < 0.05 were considered indicative of statistical significant differences between the two approaches. The sample size was based on available data.

All statistical analyses were conducted using R version 4.2.1 (R Foundation for Statistical Computing, Vienna, Austria, 2016).^1^

## Results

A total of 481 participants completed the short survey and the validated questionnaire. Of these 481 participants, 36% (*n* = 171) had MA clinical diagnosis, and 64% MO clinical diagnosis (*n* = 310). Those with MO had a higher median number of triptan use days, with one-day difference compared to those with MA (Table 1). No additional analyses were performed to explore the potential impact of this difference on diagnostic accuracy. There was no missing data.

**Table 1:** Baseline characteristics, *n* = 481 participants with both first survey and second validated questionnaire and have a clinical diagnosis of migraine with aura (MA) or migraine without aura (MO).

Characteristics	Migraine with aura	Migraine without aura	*p*-value
Number of patients, n	171	310	
Age *(years),* mean ± SD	49 (14)	51 (11)	0.098
Sex (female), n (%)	148 (87%)	264 (85%)	0.780
Chronic migraine^1^, n (%)	7 (4)	7 (2)	0.388
Monthly headache days, median ± IQR	9 [5, 14]	10 [5, 16]	0.293
Monthly migraine days, median ± IQR	5 [3, 8]	5 [3, 8]	0.356
Monthly non-migraine headache days, median ± IQR	3 [1, 8]	3 [1, 6]	0.096
Monthly acute medication days, median ± IQR	8 [4, 11]	6 [4, 10]	0.066
Monthly triptan days, median ± IQR	3 [0, 5]	4 [1, 6]	0.004
Monthly OTC^2^ medication days, median ± IQR	3 [1, 6]	3 [1, 6]	0.981

^1^Averaged over the last 3 months. ^2^OTC = over the counter medication, such as painkillers or NSAIDs. All outcomes are self-reported with a cross-sectional questionnaire, not with daily e-headache diary.

When comparing the visual aura image to clinical diagnoses as the gold standard, 35% of MO participants reported having experienced a visual aura at some point in their lives, while 11% of MA participants had not. This yielded a sensitivity of 0.89 (95%CI 0.84, 0.94), a specificity of 0.65 (95%CI 0.60, 0.70), a PPV of 0.59 (95%CI 0.52, 0.65) and a NPV of 0.92 (95%CI 0.87, 0.95). When comparing the combination of the aura image with a questionnaire, 10% of MO participants reported having experienced a visual aura, while 47% of MA participants had not. In comparison, the combination the aura image with a questionnaire resulted in a sensitivity of 0.53 (95%CI 0.45, 0.60), a specificity of 0.90 (95%CI 0.86, 0.93), a PPV of 0.74 (95%CI 0.65, 0.81) and a NPV of 0.77 (95%CI 0.73, 0.82) (Table 2).

**Table 2:** Diagnostic accuracy of visual aura image versus visual aura image combined with questionnaire, using clinical migraine diagnosis as the reference standard.

Diagnostic accuracy measures	Visual aura image	Visual aura image + questionnaire	*p*-value
Sensitivity (95%CI)	0.89 (0.84, 0.94)	0.53 (0.45, 0.60)	*<* 0.001
Specificity (95%CI)	0.65 (0.60, 0.70)	0.90 (0.86, 0.93)	< 0.001
Positive predictive value (95%CI)	0.59 (0.52, 0.65)	0.74 (0.65, 0.81)	
Negative predictive value (95%CI)	0.92 (0.87, 0.95)	0.77 (0.73, 0.82)	
Positive likelihood ratio (95%CI)	2.57 (2.33, 2.83)	5.10 (4.40, 5.91)	
Negative likelihood ratio (95%CI)	0.16 (0.10, 0.26)	0.53 (0.37, 0.76)	

CI, Confidence Interval. Comparisons of sensitivity and specificity between the two diagnostic approaches were performed using the McNemar’s chi-square test for paired proportions applied to the same cohort of participants. *P*-values indicate statistical significance of differences between the two approaches.

Among patients with MA (*n* = 171), the visual aura image alone detected 63 additional cases missed by the combination of the aura image and the questionnaire, this difference was statistically significant (McNemar Χ^2^ = 61.02, *p* < 0.001) (Supplementary Table S1). Among patients with MO (*n* = 310), the visual aura image alone produced 76 extra false positives compared to the combination of the aura image and the questionnaire this difference was statistically significant (McNemar Χ^2^ = 74.01, *p* < 0.001) (Supplementary Table S2).

## Discussion

In this cross-sectional study, we found that a single visual aura image identified most patients with migraine with aura, demonstrating high sensitivity but limited specificity. This may be acceptable for screening in the consultation room or in large-scale epidemiological or genetic studies, but it cannot reliably differentiate between patients with and without aura. Our findings highlight that adding questions on gradual spreading, positive visual phenomena, lateralization, duration of visual phenomena and temporal relationship to headache improves specificity. Combining these elements with the aura image yielded a more balanced diagnostic tool, by reducing false positives and producing a better balance of positive and negative predictive values.

This finding is consistent with previous literature that aura images may enhance diagnostic accuracy but are insufficient as a stand-alone tool (5, 9). The combination of a visual aura image with additional questionnaire, aligned with the ICHD-3 criteria and addressing gradual symptom spread over minutes, positive symptoms such as scintillations or flashes, unilaterality, aura duration and, temporal relation to headache, offers a superior approach to distinguishing MA from MO (10). Nevertheless, in large research cohorts where sensitivity is prioritized to avoid missing potential MA cases, the use of a representative image alone might be considered acceptable, though it should still be recognized that this comes at the cost of higher false-positive rates.

A key strength of this study was the availability of verified migraine diagnoses of participants by trained clinicians and a neurologist with headache expertise. Moreover, the prevalence of MA and MO in the cohort was similar to that of previous epidemiological studies, providing a realistic positive and negative predictive value (11). Furthermore, by including both web-based and clinic-based participants, we captured a broad spectrum of migraine presentations. A limitation of the study is the inclusion of only a single visual aura image, leaving unanswered whether multiple images might improve diagnostic accuracy. However, it was clearly stated that the image was only a single example, and participants were asked a broader question about their experiences with visual aura phenomena. Importantly, nearly all clinically diagnosed MA cases were identified, and presenting a larger set of images could introduce response bias (12). Another limitation is the focus on visual aura, because non-visual auras without accompanying visual symptoms are rare, this restriction is unlikely to have a substantial impact, as only 1–8% of patients in several cohorts reported non-visual aura symptoms in isolation (13–17). Finally, a potential limitation to this study is diagnostic incorporation bias. Because the physician’s ‘gold standard’ diagnosis was informed by the same validated questionnaire, the index test and the reference standard are not fully independent. This overlap may lead to an overestimation of diagnostic accuracy, particularly sensitivity. While the physician’s independent clinical judgment provides a necessary buffer, the diagnostic performances should be interpreted with this interdependence in mind.

In conclusion, presenting a single visual aura image in the consultation room or for large epidemiological or genetic studies may support the detection of migraine with aura. However, additional questioning is essential for accurate diagnosis. Particularly informative are questions on gradual symptom spread and the presence of positive phenomena, as well as unilaterality, aura duration and the temporal relation between aura and headache. These findings highlight that presenting a visual aura image to patients has limited diagnostic value on its own and that efforts to develop or distribute online iconography tools may likely be of limited clinical utility.

## Data Availability

The datasets presented in this article are not readily available because of ethical and privacy restrictions. Requests to access the datasets should be directed to g.m.terwindt@lumc.nl.

## Footnotes

1
https://www.R-project.org/
