# Supplementary material for: Diagnostic performance of one visual aura image in identifying migraine with aura
Source: Front Neurol. 2026 Mar 6;17:1774218. doi: 10.3389/fneur.2026.1774218 (PMC13003632; doi:10.3389/fneur.2026.1774218)
Supplement: Supplementary file 1 [file Data_Sheet_1.docx]

# Supplementary material

## Table S1. 2x2 table of the Visual Aura image and the Visual Aura image + extended questionnaire among participants with MA

| Visual aura image (Yes/No) | Visual aura Image + questionnaire | | |
| --- | --- | --- | --- |
|  |  | Combined method: No | Combined method: Yes |
|  | Image: No | 18 | 0 |
|  | Image: Yes | 63 | 90 |

The diagnosis migraine with aura or migraine without aura is fixed by design, as these were clinically verified diagnosis. For the combined method, “Yes” indicates the present of self-reported visual aura and “No” indicates it absence; the same coding applies for the Image variable (Yes/No).

## Table S2. 2x2 table of the Visual Aura image and the Visual Aura image + extended questionnaire among participants with MO

| Visual aura image (Yes/No) | Visual aura Image + questionnaire | | |
| --- | --- | --- | --- |
|  |  | Combined method: No | Combined methode Yes |
|  | Image: No | 202 | 0 |
|  | Image: Yes | 76 | 32 |

The diagnosis migraine with aura or migraine without aura is fixed by design, as these were clinically verified diagnosis. For the combined method, “Yes” indicates the present of self-reported visual aura and “No” indicates it absence; the same coding applies for the Image variable (Yes/No).
